# Roles for the Dorsal Striatum in Aversive Behavior

**DOI:** 10.3389/fncel.2021.634493

**Published:** 2021-02-16

**Authors:** Adrien T. Stanley, Pellegrino Lippiello, David Sulzer, Maria Concetta Miniaci

**Affiliations:** ^1^Departments of Biology and Psychiatry, Columbia University, New York, NY, United States; ^2^Department of Pharmacy, School of Medicine, University of Naples Federico II, Naples, Italy; ^3^Departments of Psychiatry, Neurology, and Pharmacology, Columbia University Medical Center, New York, NY, United States; ^4^Division of Molecular Therapeutics, New York State Psychiatric Institute, New York, NY, United States

**Keywords:** dorsal striatum, fear conditioning, inhibitory avoidance, aversion, threat

## Abstract

The ability to identify and avoid environmental stimuli that signal danger is essential to survival. Our understanding of how the brain encodes aversive behaviors has been primarily focused on roles for the amygdala, hippocampus (HIPP), prefrontal cortex, ventral midbrain, and ventral striatum. Relatively little attention has been paid to contributions from the dorsal striatum (DS) to aversive learning, despite its well-established role in stimulus-response learning. Here, we review studies exploring the role of DS in aversive learning, including different roles for the dorsomedial and dorsolateral striatum in Pavlovian fear conditioning as well as innate and inhibitory avoidance (IA) behaviors. We outline how future investigation might determine specific contributions from DS subregions, cell types, and connections that contribute to aversive behavior.

## Introduction

The ability to learn associations between environmental stimuli and aversive events is essential to survival, as it allows the organism to avoid these events and reduce the chance of harm. In a threatening context, individuals may use defense strategies, such as the fight or flight response, or passive coping, characterized by immobility and withdrawal (Koolhaas et al., 1999; Wood and Bhatnagar, 2015). However, aberrant avoidance responses can lead to psychiatric diseases, including anxiety and post-traumatic stress disorders (PTSD; Sripada et al., 2013).

Pavlovian fear conditioning paradigms in rodents have served as the most widely used approaches for the analysis of neural mechanisms of learning and memory-related to aversive stimuli (LeDoux, 2000; Maren, 2001; Cefaliello et al., 2020). During fear conditioning, animals learn to associate a neutral conditioned stimulus (CS) that can either be a temporally discrete sensory event or cues, such as a light or tone, or the environmental context itself with an aversive event such as an inescapable electric foot-shock unconditioned stimulus (US), so that the cue becomes a threatening signal and elicits conditioned reactions such as freezing and tachycardia. Both context and cue-dependent fear conditioning paradigms are learned rapidly and elicit robust memory. An aversive stimulus can also elicit actions that avoid the upcoming threat, e.g., in active avoidance conditioning, the animal learns to avoid a shock.

**Figure 1:**
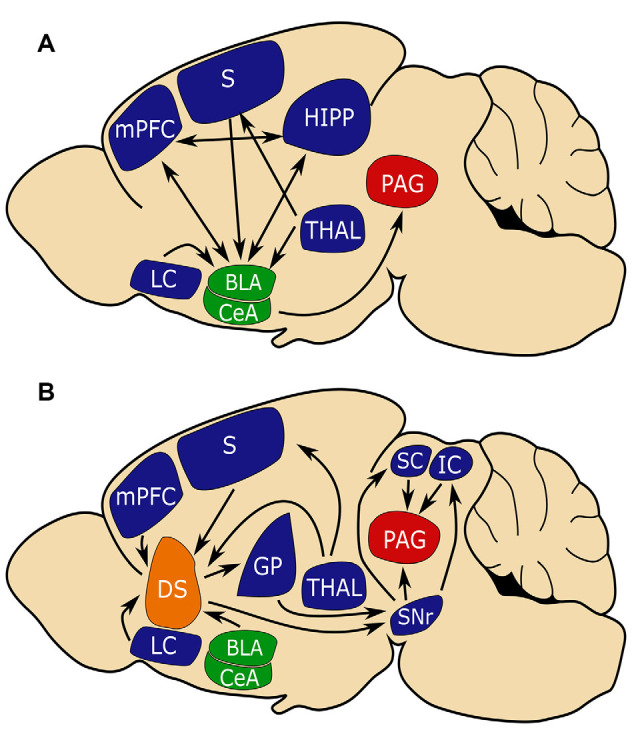
The amygdala and dorsal striatum (DS) may be similarly positioned to influence fear learning. **(A)** The conventional circuit model of fear learning with a central role for the amygdala (in green). **(B)** A circuit model that places the (DS in orange) in a similar position to the amygdala in regulating fear conditioning. Note that the amygdala and DS share inputs from the sensory cortex (S) and medial prefrontal cortex (MPFC) which is important for connecting aversive events with sensory stimuli and that both circuits converge on periaqueductal gray (PAG, in red), which is critical for mediating freezing behavior. Basal lateral amygdala (BLA), central amygdala (CEA), substantia nigra reticulata (SNR), globus pallidus (GP), superior colliculus (SC), inferior colliculus (IC), hippocampus (HIPP), thalamus (THAL), limbic cortex (LC).

Much has been learned about the neural basis of fear learning and memory through studies of fear conditioning. Studies in both humans and rodents have corroborated a central role for the amygdala in the acquisition and storage of conditioned fear (Nader et al., 2001; Phelps and LeDoux, 2005). In particular, sensory inputs from the cortex and thalamus enter the amygdala *via* the basolateral amygdala (BLA). This sensory input, as well as reciprocal input from the ventral hippocampus (HIPP) and medial prefrontal cortex (MPFC), plays an important role in encoding CS-US association. In response to CS, the BLA will activate central amygdala (CEA) nuclei which in turn sends projections to the bed nucleus of the stria terminalis, periaqueductal gray (PAG), and the hypothalamus driving the expression of behavioral and autonomic responses (LeDoux et al., 1988; Tovote et al., 2015; Krabbe et al., 2018; Sah et al., 2020; Figure 1A).

Active avoidance learning proceeds through three distinct phases, involving different neural circuits (Cain and LeDoux, 2007; LeDoux et al., 2017). The first phase consists of *CS-US fear conditioning* (i.e., the animals learn that a stimulus predicts a threat) and involves the BLA–CeA–PAG pathway mentioned above. The second phase involves *action-outcome learning* (i.e., animals learn to perform actions that terminate the CS) and depends on the activation of the basolateral amygdala-nucleus accumbens pathway; the infralimbic prefrontal cortex is also recruited to suppresses the CeA-mediated freezing and facilitate avoidance (Moscarello and LeDoux, 2013). According to LeDoux, avoidance may be reinforced not only by the removal of danger (negative reinforcement) but also by the addition of a safety cue (positive reinforcement; LeDoux et al., 2017). In the third phase, *avoidance behavior becomes habitual* (i.e., outcome independent) and likely involves the dorsal striatum (DS).

The DS has been extensively studied for its essential role in stimulus-response learning, i.e., a process by which a sensory cue can elicit a consistent motor response (Packard and Knowlton, 2002). Whether the DS contributes to the aversive learning process is not fully understood.

Various aversive stimuli such as noxious heat, cold, mechanical, and electrical stimuli alter the activity of the DS, and many studies report that alterations of DS function using lesions and microinjections affect pain perception (Chudler and Dong, 1995; Borsook et al., 2010). Interestingly, patients with Parkinson’s disease, a movement disorder mostly due to decreased dopamine release in the DS, often present with comorbid chronic pain (Buhidma et al., 2020). Chronic pain is also co-morbid with PTSD (Fishbain et al., 2017), and processing of aversive stimuli within the DS is important for pain-avoidance learning (Koyama et al., 2000).

This review highlights studies that investigate the role of the DS in aversive learning with a focus on fear conditioning and avoidance.

## Neuronal Projections from and to The Dorsal Striatum

The DS is composed of a medial portion (DMS), a lateral portion (DLS), and a posterior portion (tail of the striatum, TS), each with distinct functions and inputs (Hunnicutt et al., 2016). The DMS primarily receives inputs from limbic and association cortices such as anterior cingulate cortex, parietal association cortex, prelimbic cortex (LC), and infralimbic cortex while the DLS receives inputs primarily from the motor and somatosensory cortices such as M1/2 and S1/2. The TS primarily receives information from sensory cortices, especially the auditory and visual cortex (Haber, 2003; Hunnicutt et al., 2016). All of these regions receive dopaminergic input from the substantia nigra compacta (SNc), which plays an important role in synaptic plasticity (Arbuthnott et al., 2000). Dopaminergic projections from the lateral SNc project exclusively to the TS while all other portions of the SNc project throughout the DS (Beckstead et al., 1979).

Several regions that project to the DS subdivisions are implicated in fear learning, including glutamatergic excitatory direct projections from the BLA and mPFC, and indirectly, GABergic inhibitory inputs from the CeA (Conzales and Chesselet, 1990; Hunnicutt et al., 2016; Figure 1A). As does the amygdala, the DS receives input from the thalamus, sensory cortices, and association cortices (LeDoux, 2000; Hunnicutt et al., 2016; Figure 1B). The cortical and thalamic inputs to the amygdala are critical for associating sensory stimuli with aversive events, and it may be that similar inputs to the DS serve complementary functions (Ponvert and Jaramillo, 2019).

The DS is predominantly composed of spiny projection neurons (SPNs) that mostly express either D1 dopamine receptors or D2 receptors. D1 SPNs project directly to the substantia nigra reticulata (SNr) and are referred to as *direct pathway* SPNs. D1 SPN projections to the SNr are topographically organized with D1 SPNs of the anterior DS project medially, while D1 SPNs of the posterior DMS/DLS and TS project laterally (Hedreen and Delong, 1991). D2 SPNs indirectly project to the SNr *via* the globus pallidus (GP) externa (GPe) and subthalamic nuclei and are thus referred to as the *indirect pathway* (DeLong, 1990; Bertran-Gonzalez et al., 2010). D2 SPN projection to the GPe is also topographically organized with D2 SPNs of anterior DS projecting anteriorly, while D2 SPNs of the posterior DS project posteriorly (Hedreen and Delong, 1991).

Activation of the two SPN populations often elicits opposite effects on behavior (Lenz and Lobo, 2013). Both the GPe and SNr have been shown to play roles in aversive behaviors (Ipser et al., 2013; Hormigo et al., 2016; Almada et al., 2018). The SNr projects to several regions involved in the fear response including the superior colliculus (SC), inferior colliculus (IC), and PAG (Castellan-Baldan et al., 2006). Together, these pathways place the DS in a position to integrate sensory and aversive information as well as modulate aversive responses.

## Dorsal Striatum and Fear Conditioning

Both cued fear and contextual fear conditionings are composed of three distinct phases: acquisition, consolidation, and memory recall.

**Figure 2:**
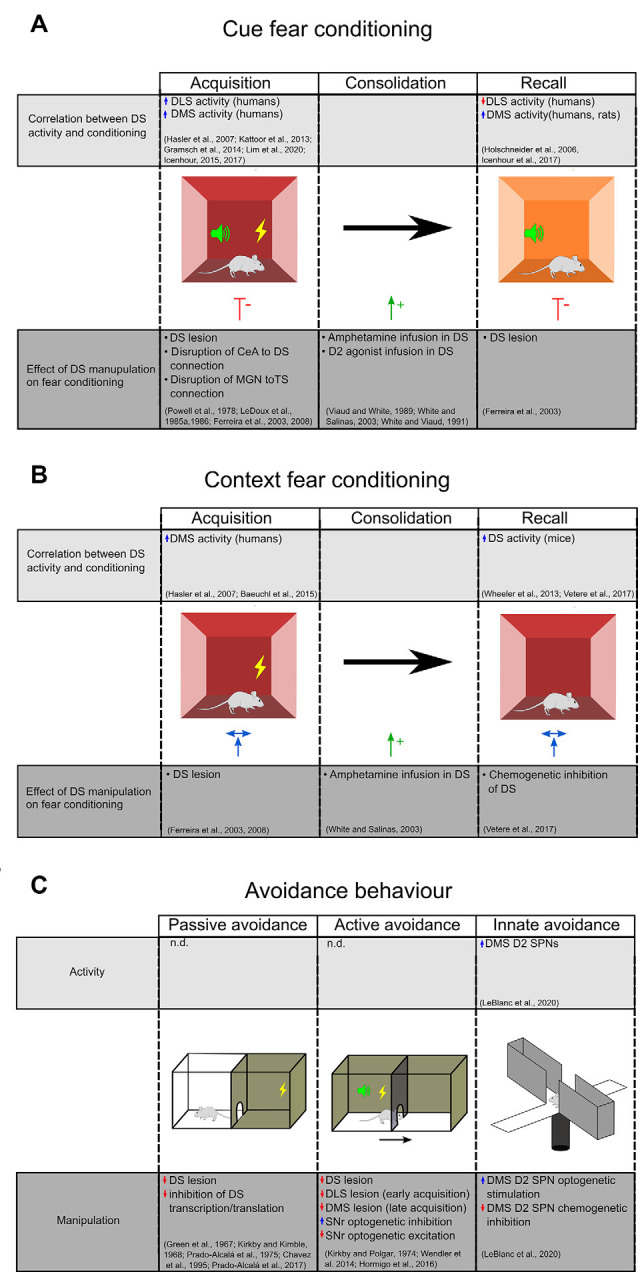
Evidence for DS’s role in aversive behavior. **(A)** The upper chart highlights studies that demonstrate a correlation between DS activity during different stages of cue fear conditioning. The lower chart highlights studies on the effect of DS manipulations on cue fear learning. **(B)** Studies examining the effect of context fear conditioning on DS activity (top) and the effect of DS manipulations on context fear learning (bottom). Central amygdala (CEA), medial geniculate nucleus (MGN), dorsal striatum (DS), dorsal medial striatum (DMS), dorsolateral striatum (DLS), the tail of the striatum (TS). **(C)** Evidence for DS’s role in inhibitory and innate avoidance. Dorsal striatum (DS), dorsal medial striatum (DMS), dorsolateral striatum (DLS), substantia nigra reticulata (SNR).

Brain regions involved in fear conditioning are differentially engaged by cued vs. contextual responses, as well as during different stages of the conditioning process. For example, the hippocampus has been shown to play a pivotal role in the acquisition and consolidation of contextual fear learning with little involvement in cued fear (Sanders et al., 2003), while the amygdala plays roles in both forms and during all phases of fear conditioning (LeDoux, 2000; Tovote et al., 2015). A summary of activity changes and the consequences of manipulating the DS function are outlined in Figures 2A,B.

## Cued Fear Conditioning

Cued fear conditioning paradigms involve placing an animal in a chamber with metal bar floors, and after a habituation period, presenting the mouse an auditory CS and an electrical foot-shock (US) that temporally overlaps with the CS. To test the strength of the CS-US association, during the recall phase, the mouse is placed in a new context and freezing responses to the CS are measured.

Functional magnetic resonance imaging and positron emission tomography (PET) studies in humans demonstrate that the putamen (corresponding to the mouse DLS), but not caudate (corresponding to rodent DMS), is activated during the acquisition phase of fear conditioning (Hasler et al., 2007; Kattoor et al., 2013; Gramsch et al., 2014). However, further studies in humans show that the caudate is also active during both acquisition and recall (Icenhour et al., 2015, 2017). An increase in DMS activity has also been demonstrated in rats during cued fear recall, in association with a decrease in the DLS blood flow (Holschneider et al., 2006). Based on these studies, we may argue that there is a differential contribution of DLS and DMS in fear acquisition and recall but conflicts remain to be addressed.

To determine the causal relationship between striatal activity and cued fear learning, selective lesions and pharmacological inactivation studies have been performed. For example, in rats, blocking DS function by an electrolytic lesion or inhibition with the GABA_A_ agonist, muscimol, before training or before the recall, reduced freezing responses to the cue (Ferreira et al., 2003, 2008). Similarly, lesion of the DS in rabbits impairs corneo-retinal responses to learned fear cues (Powell et al., 1978).

At odds with the results of studies indicating a role for DS conditioning in fear learning is a study that compared the effect of selective lesions to the DLS, DMS, or ventral striatum on cued fear conditioning in rats, that concluded that only lesions to the ventral striatum decreased freezing during training and recall (Wendler et al., 2014). We note however that rather than measuring freezing during CS presentation, the investigators measured freezing during the entire recall session which took place in the same context as the training session, and so did not separate freezing response generated by context from that of the cue.

More broadly, a major caveat of brain lesioning studies is that they do not disentangle the contributions of the DS to acquisition vs. consolidation or recall, as these manipulations affect all phases of fear conditioning. A related issue with classical lesion studies is that they indiscriminately destroy all of the cells in a region of the DS, and so cannot identify neuronal populations that serve different or even opposing functions during fear conditioning.

Pharmacological manipulations also implicate the DS in cued fear conditioning. Selective infusion of amphetamine, an agent that drives the release of dopamine and other monoamines, during the consolidation phase enhanced fear responses during recall (Viaud and White, 1989; White and Salinas, 2003). This effect was replicated with an infusion of a D2 agonist, but not a D1 agonist, into the DS (White and Viaud, 1991). These results suggest a differential involvement of D1 and D2 receptors in cued fear consolidation. Since D2 receptors are expressed on SPNs and GABAergic and cholinergic interneurons as well as dopaminergic afferents (Fisher et al., 1994), it will be important to examine the contributions of these cell types on fear consolidation.

Disrupting the connections between DS and the other brain areas involved in fear learning can also influence fear response. Studies in rats have examined the consequences of severing the connections between the CeA and DS in fear learning (Ferreira et al., 2008). Because the CeA connects to the DS *via* an indirect and exclusively ipsilateral projection to the SNc (Conzales and Chesselet, 1990), the authors performed asymmetrical lesions, consisting of a unilateral CeA lesion combined with a contralateral DS lesion, while leaving the contralateral sides functional. They found that this manipulation before training, but not before recall, impaired the freezing response.

The same ablation approach in rats revealed that projections from the medial geniculate nucleus (MGN) of the thalamus to TS are important for cued fear learning (LeDoux et al., 1985a, 1986). The MGN is a portion of the thalamus that relays auditory information from the IC to the TS as well as the amygdala and auditory cortex (LeDoux et al., 1984, 1985b) Bilateral lesion of MGN before training impairs freezing during recall. This effect is replicated by unilateral ablation of MGN combined with the contralateral lesion of TS or amygdala (LeDoux et al., 1985a) These results suggest that auditory information from MGN to the TS/amygdala is essential for cued fear learning.

What role is the DS playing in cued fear learning? These studies have shown that DS activity and cued fear learning are correlated and causally related. However, because studies exploring the causal relationship rely on lesions and pharmacological manipulations that affect many subregions and cell types within the DS, it has been difficult to determine the role of the DS. Further investigations using techniques with cell-specific and region-specific targeting are needed to strengthen the evidence of the causal relation of DS activity and cued fear learning.

## Contextual Fear Conditioning

Contextual fear conditioning paradigms consist of placing an animal in a chamber with metal bar floors where it is administered an electric foot-shock. In this paradigm, the foot-shock is a US and the environmental context of the conditioning chamber is the CS. To test the degree of CS-US association, during the recall phase, mice are returned to the conditioning chamber and the time spent freezing is recorded.

PET imaging in humans using [O-15]H_2_O has revealed that the caudate is activated during the acquisition of contextual fear conditioning (Hasler et al., 2007; Baeuchl et al., 2015). A role for the DS is further supported by experiments in mice, where recall of contextual fear has been shown to increase DS expression of the immediate-early gene cFos, an indicator of neural activity (Wheeler et al., 2013; Vetere et al., 2017). In contrast, rats that naturally have a low response to contextual fear conditioning have higher mRNA expression of striatal NMDA subunit NR2A (Schenberg et al., 2006); given a requirement of NR2A for long term potentiation in the DS (Li et al., 2009), this finding suggests that enhanced plasticity within the DS may attenuate contextual fear learning. Studies investigating the causal relationship of DS activity and plasticity on contextual fear learning may shed light on this issue.

Studies selectively ablating the DS before training show no effect on contextual fear learning (Ferreira et al., 2008). Similarly, a study using chemogenetic inhibition of DS after contextual fear training showed no effect on freezing during recall (Vetere et al., 2017). On the other hand, infusion of amphetamine selectively into the DS of rats during the consolidation phase enhances the freezing during recall of contextual fear (White and Salinas, 2003), suggesting that dopamine in the DS promotes consolidation of contextual fear. How the dopamine, and eventually the D1 and D2 SPNs are involved in contextual fear learning remains an open question.

While these studies indicate that the DS may play a selective role in the consolidation of contextual fear, controversies remain and there are many gaps in our knowledge. Additional studies are, therefore, required that target the DS during acquisition and recall.

## Dorsal Striatum and Avoidance Behavior

In the inhibitory avoidance (IA) test, animals learn to avoid an environment where they have previously been exposed to an aversive stimulus (US, such as a foot-shock) by remaining in the brightly lit side of a two-compartment chamber (passive avoidance paradigm) or by running into the opposite side compartment following a CS presentation (active avoidance paradigm). In this paradigm, the animals have to suppress their innate avoidance behavior i.e., their tendency to avoid bright and open areas.

A role for the DS in IA has been known since the 1960s. Several studies in rodents demonstrated that electrolytic or chemical disruption of the caudate nucleus impairs the acquisition and the retention of the passive IA task (Green et al., 1967; Kirkby and Kimble, 1968; Prado-Alcalá et al., 1975; Chavez et al., 1995; Figure 2C). A marked deficit of consolidation of a passive IA task has also been observed following inhibition of transcription and translation in DS (Prado-Alcalá et al., 2017). Interestingly, while foot-shock alone does not change the numbers of dendritic spines of SPNs in the DLS and DMS, intense IA training induces a significant increase in spine density that is proportional to the foot-shock intensity. The learning-induced increase of dendritic spines in DS may represent a cellular mechanism underlying the consolidation and persistence of memory storage (Bello-Medina et al., 2016).

Lesion studies have also demonstrated that DS has an important role in the active avoidance task (Kirkby and Polgar, 1974; Figure 2C). Wendler et al. (2014) have demonstrated a differential involvement of DS subregions since lesions of DLS impaired early acquisition whereas DMS lesion impaired the late phases of learning and the extinction. The precise striatal circuit that results in active avoidance behavior remains elusive. Using chemogenetics and optogenetics, Hormigo and collaborators (Hormigo et al., 2016) recently found that the SNr, the main DS output, modulates the avoidance response to the CS. Indeed, inhibition of SNr firing facilitates active avoidance during tone presentation while SNr excitation blocks the response. According to their putative circuit model, the CS-driven avoidance behavior may engage a multisynaptic loop that originates within and returns to the superior colliculus *via* the SNr (Hormigo et al., 2016). The SC detects sensory stimuli and sends information to the striatum *via* the posterior intralaminar nucleus (Linke, 1999; Krout et al., 2001). Once activated by CS, the striatal direct pathway SPNs may suppress the SNr and consequently disinhibit its projections to the SC and the mesencephalic locomotor central pattern generators (MacKay-Lyons, 2002), thus promoting active avoidance behavior.

Elevated plus maze and open field are commonly used to assess the innate tendency of mice to avoid brightly lit and open spaces such as the open arms of a maze or the center area of an open field. Evidence is now emerging that the DMS, and in particular the D2R-expressing SPNs, plays an important role in innate avoidance behavior in the elevated plus-maze and open-field behavioral tests (Figure 2C). Using fiber photometry, LeBlanc et al. (2020) demonstrated that D2 SPNs activity increases as mice enter anxiogenic areas of these tasks. Also, they demonstrated that optogenetic stimulation of D2 SPNs promotes avoidance of open areas in both tasks, while the chemogenetic inhibition of D2 SPNs reduces avoidance behavior. Interestingly, knocking out the D2R on cholinergic interneurons or dopamine neurons did not affect the mice’s performance in the exploratory tasks, suggesting that the DMS indirect pathway neurons are critical for evoking aversive behavior.

## Conclusions and Directions for Future Research

While it is well established that the DS facilitates reward-based stimulus-response learning (Packard and Knowlton, 2002), there is far less evidence that this region mediates the freezing or escape responses to aversive stimuli. Our review of current data indicates that the patterns for appetitive learning are different from those associated with aversive instrumental conditioning and strongly supports a role for the DS in cued fear conditioning and avoidance learning, while there are conflicting findings of its involvement in contextual fear.

A fundamental difference between the learning paradigms seems to reflect the participation of different DS regions. In appetitive instrumental conditioning, the goal-directed action is quickly acquired and involves DMS activation, while the habit component is learned slowly and is apparently mediated by the DLS (Yin et al., 2006). This suggests that the DMS is required for the early stages of appetitive learning, while the DLS is involved in later stages.

Aversive learning appears to engage DS subregions in a different manner than appetitive learning. In active avoidance, DLS lesions selectively impair the early stage of acquisition of an active avoidance task, while DMS lesions selectively impair the late stage of learning (Wendler et al., 2014). Similarly, in fear conditioning, DMS appears to be particularly involved in late acquisition whereas DLS is engaged during both the early and late stages of acquisition (Icenhour et al., 2015).

To explain the differential involvement of DS subregions in aversive learning vs. appetitive learning, we propose the following hypotheses: (1) the DLS may work together with the amygdala to develop cue-outcome associations. With extensive learning, avoidance behaviors may shift to habitual defensive behaviors, which might be under the control of the DMS. (2) The ability of DMS D2 SPNs to respond and drive innate avoidance in the elevated plus-maze and open field (LeBlanc et al., 2020) suggests that DMS activity is required for innate avoidance behavior. DMS D2 SPN activity may also play an important role in conditioned avoidance behavior and, with extensive training, becomes engaged as the animal learns successful avoidance strategies.

These hypotheses remain speculative, and to test them, future studies must compare how selective interference with DMS and DLS pathways influence revaluation, the ability to change the outcome value of a CS in an aversive paradigm (Campese et al., 2019). Since changes in outcome value alter behavior when actions are goal-directed but not habit directed, interference with the DMS and DLS should alter performance if they mediate goal-directed action, and not habit action. Overall, the role of specific inputs to these DS regions and the differences between direct and indirect SPN outputs from each are poorly understood in aversive learning. The adaption of chemogenetic and optogenetic techniques that selectively target or report from specific cell populations in DS subregions during the different phases of fear conditioning and avoidance behaviors will aid in understanding the DS contribution to aversive learning.

## Author Contributions

MCM and DS designed the outline of the article. ATS, PL, MCM, and DS wrote the manuscript. ATS and PL prepared the figures. All authors contributed to the article and approved the submitted version.

## Conflict of Interest

The authors declare that the research was conducted in the absence of any commercial or financial relationships that could be construed as a potential conflict of interest.
